# Mogroside V Alleviates Oocyte Meiotic Defects and Quality Deterioration in Benzo(a)pyrene-Exposed Mice

**DOI:** 10.3389/fphar.2021.722779

**Published:** 2021-08-26

**Authors:** Lumin Sui, Ke Yan, Huiting Zhang, Junyu Nie, Xiaogan Yang, Chang-Long Xu, Xingwei Liang

**Affiliations:** ^1^ State Key Laboratory for Conservation and Utilization of Subtropical Agro-bioresources, College of Animal Science and Technology, Guangxi University, Nanning, China; ^2^ Reproductive Medical Center Nanning Second People’s Hospital, Nanning, China

**Keywords:** benzo(a)pyrene, mogroside V, Siraitia grosvenorii, oocyte maturation, embryo development, oxidative stress

## Abstract

Accumulating evidence has demonstrated that benzo(a)pyrene (BaP) exposure adversely affects female reproduction, especially oocyte meiotic maturation and subsequent embryo development. Although we previously found that mogroside V (MV), a major bioactive component of *S. grosvenorii*, can protect oocytes from quality deterioration caused by certain stresses, whether MV can alleviate BaP exposure-mediated oocyte meiotic defects remains unknown. In this study, female mice were exposed to BaP and treated concomitantly with MV by gavage. We found that BaP exposure reduced the oocyte maturation rate and blastocyst formation rate, which was associated with increased abnormalities in spindle formation and chromosome alignment, reduced acetylated tubulin levels, damaged actin polymerization and reduced Juno levels, indicating that BaP exposure results in oocyte nucleic and cytoplasmic damage. Interestingly, MV treatment significantly alleviated all the BaP exposure-mediated defects mentioned above, indicating that MV can protect oocytes from BaP exposure-mediated nucleic and cytoplasmic damage. Additionally, BaP exposure increased intracellular ROS levels, meanwhile induced DNA damage and early apoptosis in oocytes, but MV treatment ameliorated these defective parameters, therefore it is possible that MV restored BaP-mediated oocyte defects by reducing oxidative stress. In summary, our findings demonstrate that MV might alleviate oocyte meiotic defects and quality deterioration in BaP-exposed mice.

## Introduction

With the acceleration of urbanization and industrialization, air pollution is becoming an adverse environmental factor that threatens human health (Vineis and Husgafvel-Pursiainen, 2005). Polycyclic aromatic hydrocarbons (PAHs) are potent contaminants of concern in haze (Idowu et al., 2019). PAHs contain a variety of detrimental chemicals, of which benzo(a)pyrene (BaP) is prominent, occupying approximately 50% of their carcinogenic potential. BaP is produced by incomplete combustion of organic materials such as coal, petroleum, wood and organic polymers (Qi et al., 2019). Additionally, it is released from wildfire, smoking, cooking and other sources of environmental smoke (Jorgensen et al., 2013; Jeffery et al., 2018). Hence, BaP exists ubiquitously in air, water and soil (Sverdrup et al., 2002; Baklanov et al., 2006; Lohmann et al., 2009; Sun et al., 2017) and can be easily absorbed into the human body by inhalation, ingestion and skin contact (Binnemann, 1979; Qi et al., 2017; Lao et al., 2020). Due to its widespread existence and toxicity to human and animal health, BaP has received increasing attention.

Accumulating evidence has demonstrated that BaP exposure reduces female reproductive performance. For humans, the BaP concentration in the serum and follicular fluid of smokers is higher than that of nonsmokers (Neal et al., 2008). In animal models, BaP exposure adversely impacts follicle growth, ovulation, and corpora luteal formation and further inhibits oocyte meiotic maturation, compromises embryo development competence and impairs female fertility in mice (Swartz and Mattison, 1985; Sobinoff et al., 2012; Zhang et al., 2018a; Liao et al., 2020). *In vitro* experiments further confirmed that culture medium supplemented with BaP causes porcine oocyte meiotic arrest (Miao et al., 2018). Unexpectedly, we observed that female mouse exposure to BaP even led to a reduction in oocyte meiotic maturation and early embryonic development in the resulting offspring (Sui et al., 2020). Although BaP is a toxicant to oocytes, certain chemicals, such as melatonin and ginsenoside compound K, might prevent oocytes from BaP exposure-mediated defects (Miao et al., 2018; Luo et al., 2020).

Mogrosides are the most abundant bioactive components of *S. grosvenorii* (also known as monk fruit and luo han guo) and have diverse functions, including antioxidant (Chen et al., 2007; Harada et al., 2016), anti-inflammatory (Di et al., 2011), antidiabetic (Qi et al., 2008), antitumour and anticarcinogenic functions (Liu et al., 2016a; Liu et al., 2016b). Mogroside V (MV) is the major component of mogrosides in the fruit of *S. grosvenorii*. *In vitro* studies have demonstrated that MV can alleviate lipopolysaccharide-induced inflammation in cultured cells (Li et al., 2019; Liu et al., 2021). Moreover, we found that MV supplementation in *vitro* maturation medium can promote porcine oocyte maturation (Nie et al., 2020), alleviate the quality deterioration of *in vitro* ageing oocytes (Nie et al., 2019), and protect porcine oocytes from meiotic defects induced by LPS (Yan et al., 2021). Due to its beneficial effects on oocyte maturation and competence, we speculate that MV can protect oocytes from meiotic defects caused by BaP exposure.

In this study, to determine how MV alleviates BaP exposure-mediated oocyte meiotic defects, female mice were administered BaP and MV. Then, we examined the effects of MV on first polar body extrusion, early embryonic development, cytoskeletal structure, Juno content, reactive oxygen species (ROS) level, DNA damage and apoptosis level in oocytes. This study can help us to understand the protective roles of MV on BaP-exposed oocytes.

## Materials and Methods

### Reagents

Chemicals and reagents were purchased from Sigma Chemical Company (St. Louis, MO, United States), unless otherwise stated.

### Animals

All experiments were approved by the Institutional Animal Care and Use Committee (IACUC) of Guangxi University in Nanning and conducted in accordance with the National Institutes of Health Guidelines for the Care and Use of Laboratory Animals. All efforts were made to minimize animal suffering. Four-to six-week-old female Institute for Cancer Research (ICR) mice were obtained from Guangxi Medical University and housed in filter cages in a conventional animal house at controlled temperature and humidity with a 12 h light: 12 h dark cycle.

### BaP and MV Treatment

Female mice were gavaged daily with 20 mg/kg body weight BaP and 10 mg/kg, 20 mg/kg, 40 mg/kg body weight MV (Chengdu Biopurify Phytochemicals Ltd. China) at 10 AM for 10 continuous days. The doses of BaP and MV were selected based on previous reports (Liu et al., 2016b; Zhang et al., 2018b). The mice that only received corn oil were named the “Con group”, received BaP were named the “BaP group”, and received BaP and MV were named the “BaP + MV group”.

### *In vitro* Maturation and Superovulation

Immature oocytes were obtained from ovaries and cultured in M16 medium for 13 h, when the first polar body extrusion (PBE) rate was examined. On the evening of the final day of treatment, female mice were superovulated by peritoneal injection of 10 IU pregnant mare serum gonadotropin (PMSG, Ningbo Second Hormone Factory, Ningbo, China) followed by an injection of 10 IU human chorionic gonadotropin (HCG, Ningbo Second Hormone Factory) in 48 h intervals. To obtain MII oocytes, mice were euthanized by cervical dislocation 13–14 h after HCG injection. Then, the oviducts were dissected and oocytes were released from the oviduct ampulla into M2 medium.

### *In vitro* Fertilization

Sperm were obtained from the caudal epididymis of 16-weeks-old male mice and capacitated in human tubal fluid (HTF) medium for 1 h at 37°C and 5% CO_2_. Then, sperm were added to a 200 µl HTF fertilization drop containing ovulated oocytes. The sperm concentration for fertilization was 2 × 10^6^/ml. After 5 h, the oocytes were removed from the fertilization drop into potassium-supplemented simplex optimized medium (KSOM) for continuous incubation. Two-cell-stage and blastocyst-stage embryos were examined at 24 and 96 h after culture, respectively.

### Immunofluorescence Staining and Microscopy

First, MII oocytes were collected and washed with phosphate-buffered saline (PBS) containing 0.1% polyvinyl alcohol (PVA) and fixed with 4% paraformaldehyde (PFA) at room temperature (RT) for 30 min. Then, oocytes were permeabilized with 0.5% Triton X-100 for 20 min followed by a block with 1% BSA in PBS for 1 h. Next, oocytes were incubated with primary antibodies, anti-α-tubulin-fluorescein isothiocyanate (FITC) antibody (Invitrogen, United States, 1:300), phalloidin-FITC antibody (Sigma, United States, 1:300), and anti-gamma H2A.X antibody (Abcam, United Kingdom, 1:300) and FITC anti-mouse FR4 (Biolegend, United States, 1:100) overnight at 4°C. After that, oocytes were washed and incubated with appropriate secondary antibody (ZSGB-BIO, Beijing, China) for 1 h at RT. Oocytes were washed thoroughly and then stained with 10 μg/ml Hoechst 33,342 for 10 min. Finally, the oocytes were mounted on glass slides, and images were captured under a confocal laser scanning microscope (TCS-SP8MP, Leica, Germany).

### Detection of ROS Level

To determine the intracellular ROS levels, oocytes were washed and then incubated with 10 µM dichlorodihydrofluorescein diacetate (DCFH-DA; Beyotime Institute of Biotechnology) for 30 min at 37°C. Next, oocytes were washed, and images were captured under a fluorescence microscope (Nikon, Japan).

### Annexin-V Staining

The levels of early apoptosis were examined by an Annexin-V assay kit (Beyotime Institute of Biotechnology). Oocytes were washed and stained with 90 µl of binding buffer containing 10 μl of Annexin-V-FITC for 10 min in the dark. The oocytes were mounted on glass slides, and images were captured by a confocal laser scanning microscope.

### Statistical Analysis

At least triplicate was performed for each analysis and at least three mice were used for each repetition. Data are expressed as the means ± SEM. Prism seven software (GraphPad, San Diego, CA, United States) was used to analyse the data by one-way ANOVA. The fluorescence intensity was calculated by using NIH ImageJ software. The level of significance was accepted as *p* < 0.05.

## Results

### MV Alleviates the Inhibition of Oocyte Meiotic Maturation and Embryonic Development in BaP-Exposed Mice

**FIGURE 1:**
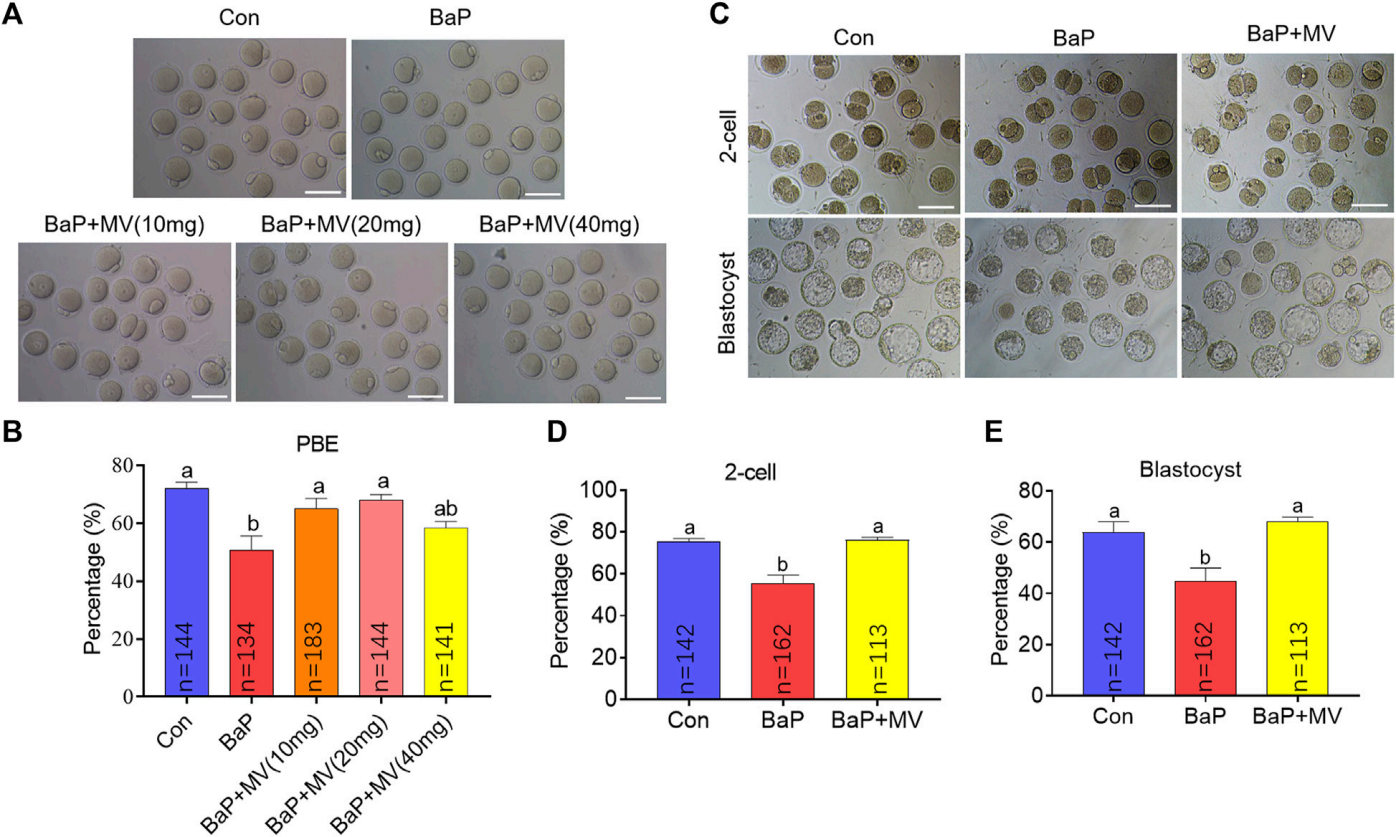
Effects of MV on meiotic maturation and embryo development in BaP-exposed mice. Oocytes were matured *in vitro* to examine meiotic progression. **(A)** Representative images of oocytes at 13 h of *in vitro* maturation in different groups. **(B)** The rates of first polar body extrusion (PBE) in the Con, BaP, BaP + MV (10 mg), BaP + MV (20 mg), and BaP + MV (40 mg) groups. BaP, Benzo(a)pyrene. MV, mogroside V. *In vitro* fertilization was performed to determine oocyte developmental competence. **(C)** Representative images of 2-cell embryos and blastocysts in the Con, BaP and BaP + MV groups. **(D)** Percentage of 2-cells in the Con, BaP and BaP + MV groups. **(E)** Percentage of blastocysts in the Con, BaP and BaP + MV groups. Scale bar = 100 μm. The data are presented as the mean ± SEM. ^a-b^Means not sharing a common superscript are significantly different (*p* < 0.05).

It has been demonstrated that 20 mg/kg body weight BaP exposure inhibits oocyte maturation; therefore, this dose was used in our study. Concomitantly, the mice were treated with different concentrations of MV (0, 10, 20, and 40 mg/kg body weight). As shown in Figures 1A,B, the first polar body extrusion (PBE) rate of oocytes in the BaP group was significantly lower than that in the Con group (*p* < 0.05), suggesting that oocyte maturation is inhibited by maternal BaP exposure. After treatment with MV, the PBE rates of the BaP + MV (10 and 20 mg/kg) groups were higher than those of the BaP group (*p* < 0.05), but there was no difference between the BaP and BaP + MV (40 mg/kg) groups (*p* > 0.05) (Con 72.00 ± 2.20% *vs* BaP 50.73 ± 4.90% *vs* BaP + MV (10) 62.66 ± 3.90% *vs* BaP + MV (20) 68.00 ± 1.94% *vs* BaP + MV (40) 58.44 ± 2.20%). Therefore, 20 mg/kg body weight MV was used for subsequent experiments.

Because embryonic development potential is a fundamental indicator of oocyte cytoplasmic maturation, we next assayed whether MV could alleviate the inhibition of embryo development caused by BaP exposure. Ovulated oocytes of the Con, BaP and BaP + MV groups were used to generate zygotes through IVF. As shown in Figures 1C–E, the percentages of 2-cell embryos and blastocysts were significantly lower in the BaP group than in the Con group (*p* < 0.01), suggesting that BaP exposure decreases early embryonic development. However, the percentages of 2-cell and blastocysts in the BaP + MV group were significantly higher than those in the BaP group (*p* < 0.01) and were not different from those in the Con group (2-cell: Con 75.45 ± 1.41% *vs* BaP 55.54 ± 3.90% *vs* BaP + MV 76.09 ± 1.26%; blastocyst: Con 63.77 ± 4.19% *vs* BaP 44.63 ± 5.25% *vs* BaP + MV 68.12 ± 1.65%). Taken together, the above data showed that BaP exposure inhibits oocyte maturation and compromises early embryonic development.

### MV Ameliorates Abnormalities in Spindle Assembly, Chromosome Alignment and α-tubulin Acetylation in Oocytes of BaP-Exposed Mice

**FIGURE 2:**
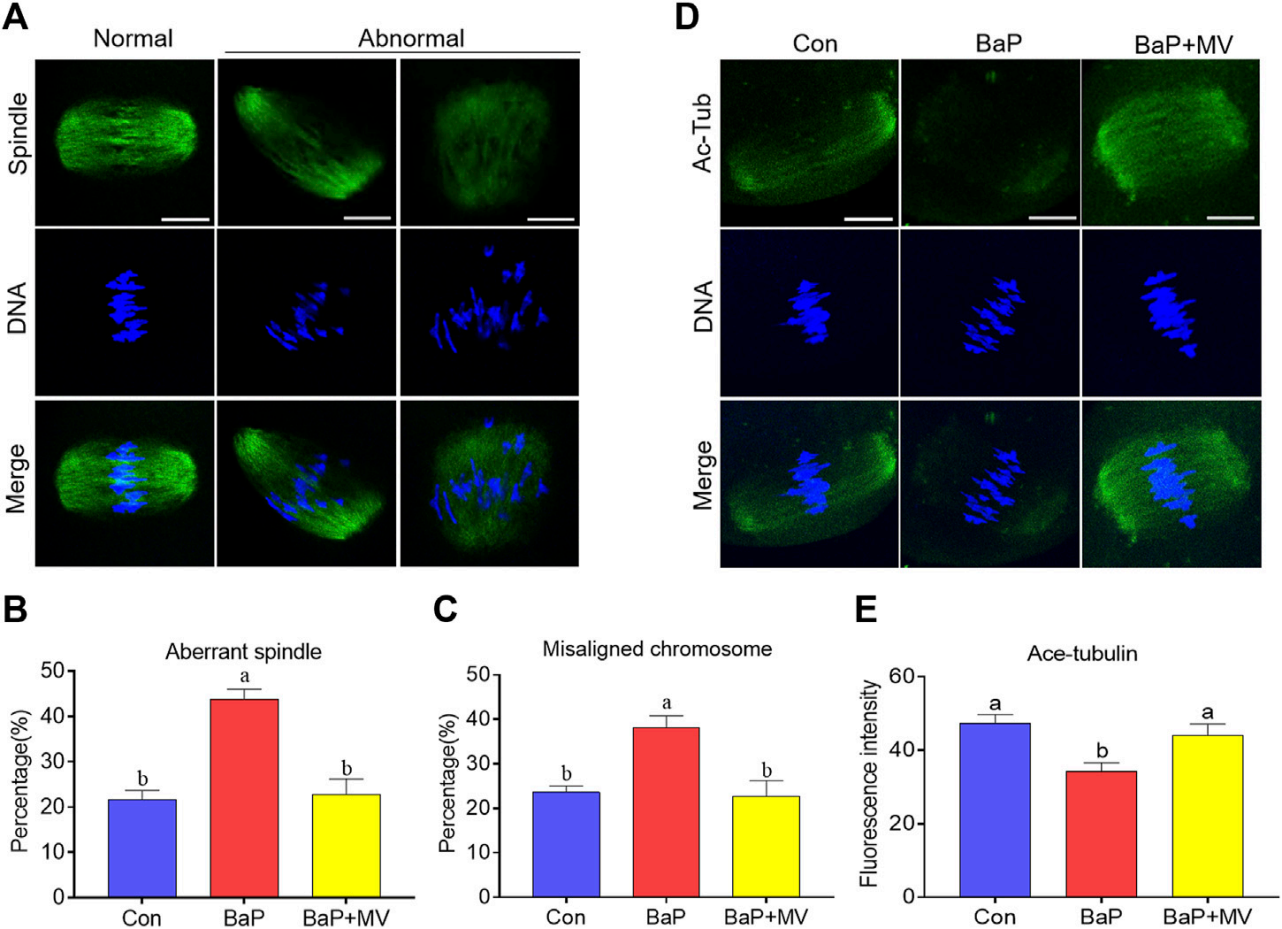
Effects of MV on spindle assembly, chromosome alignment and α-tubulin acetylation in the oocytes of BaP-exposed mice. **(A)** Representative images of spindle morphology and chromosome alignment in oocytes. **(B)** Quantification of the disorganized spindle rates in the Con, BaP and BaP + MV groups. **(C)** Quantification of the misaligned chromosome rates in the Con, BaP and BaP + MV groups. **(D)** Representative images of acetylated α-tubulin (Ac-Tub) in Con, BaP and BaP + MV oocytes. **(E)** Quantification of the fluorescence intensity of acetylated α-tubulin in the Con, BaP and BaP + MV groups. Scale bar = 25 μm. The data are presented as the mean ± SEM. ^a-b^Means not sharing a common superscript are significantly different (*p* < 0.05).

Oocytes were stained with an anti-α-tubulin antibody and Hoechst 33,342 to observe spindle assembly and chromosome alignment, respectively. As shown in Figures 2A,B higher rate of aberrant spindles was observed in BaP-exposed oocytes than in Con-exposed oocytes (Con 21.67 ± 2.04% *n* = 76 *vs* BaP 43.77 ± 2.27% *n* = 71, *p* < 0.01), but there was a significant reduction in the BaP + MV group compared to the BaP group (BaP 43.77 ± 2.27% *n* = 71*vs* BaP + MV 22.77 ± 3.44% *n* = 78, *p* < 0.01). Similarly, as shown in Figures 2A,C higher rate of misaligned chromosomes was detected in the BaP group (Con 23.61 ± 1.39% *n* = 76 *vs* BaP 38.09 ± 2.71% *n* = 71, *p* < 0.05), and MV reduced the misaligned chromosomes in BaP-exposed oocytes (BaP 38.09 ± 2.71% *n* = 71 *vs* BaP + MV 22.77 ± 3.44% *n* = 78, *p* < 0.05). These results showed that MV can alleviate abnormalities in spindle organization and chromosome alignment in oocytes of BaP-exposed mice.

Because acetylated α-tubulin is essential for spindle formation, we next examined the acetylation level of α-tubulin by an anti-acetyl-α-tubulin antibody. As shown in Figures 2D,E, we observed that BaP-exposed group oocytes had significantly reduced signals of acetylated α-tubulin compared to the Con group (Con 47.28 ± 2.47 *n* = 41 *vs* BaP 34.14 ± 2.49 *n* = 40, *p* < 0.01), but the BaP + MV group had increased acetylated α-tubulin levels compared to the BaP group (BaP 34.14 ± 2.49 *n* = 40 *vs* BaP + MV 44.03 ± 3.14 *n* = 40, *p* < 0.01). This suggests that MV restores the acetylation level of α-tubulin in oocytes of BaP-exposed mice.

### MV Restores Polymerization of Actin in Oocytes of BaP-Exposed Mice

**FIGURE 3:**
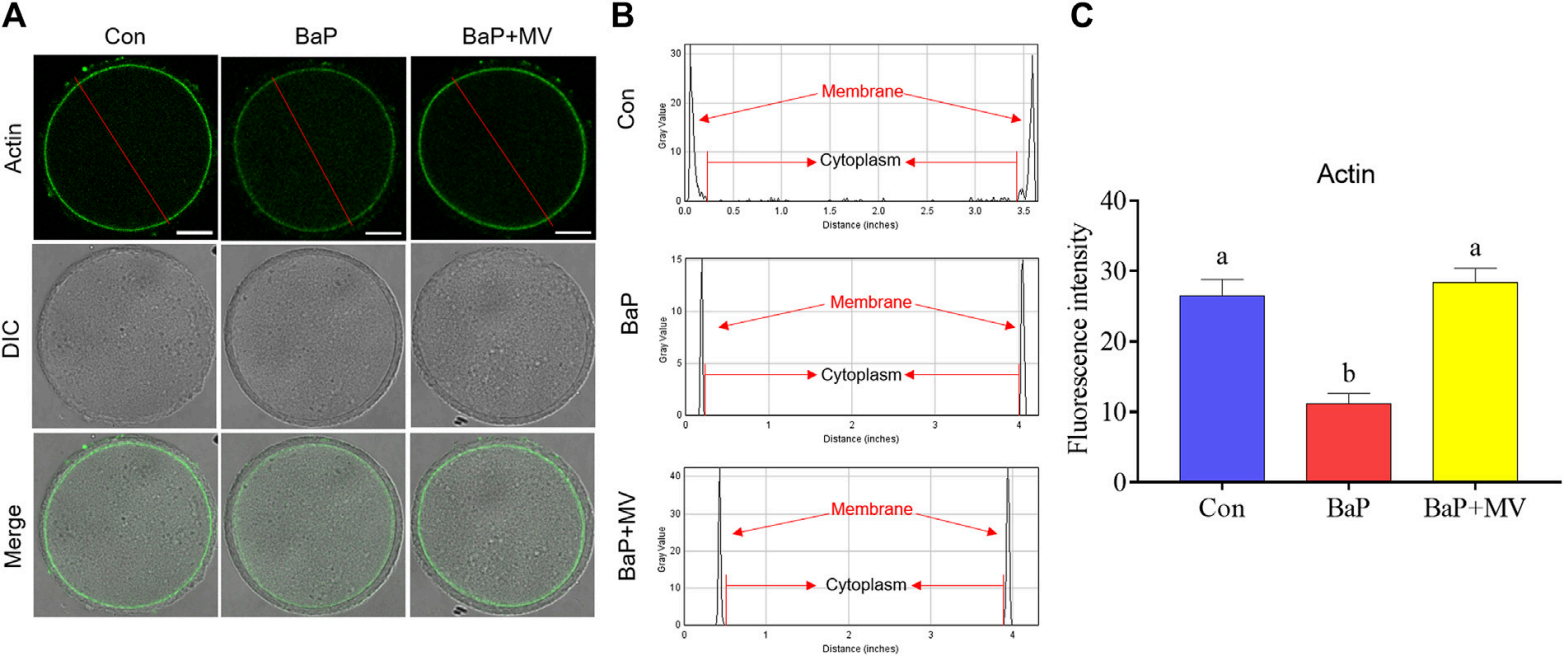
Effects of MV on actin dynamics in the oocytes of BaP-exposed mice. **(A)** Representative images of actin filaments in Con, BaP and BaP + MV oocytes. **(B)** The distribution of actin fluorescence signals in the oocyte membrane cytoplasm. **(C)** The fluorescence intensity of actin signals in the Con, BaP and BaP + MV groups. Scale bar = 25 μm. The data are presented as the mean ± SEM. ^a-b^Means not sharing a common superscript are significantly different (*p* < 0.05).

To investigate the effects of MV on actin filaments in oocytes of BaP-exposed mice, phalloidin-TRITC was used to label F-actin. As shown in Figure 3A, oocytes in the Con and BaP + MV groups had strong signals on the plasma membrane, but oocytes in the BaP group had weak signals. Quantitative analysis of fluorescence intensity showed that actin signals were significantly decreased due to BaP exposure (Con 26.50 ± 2.29% *n* = 40 *vs* BaP 11.23 ± 1.41% *n* = 37, *p* < 0.0001), but increased after treatment with MV (BaP 11.23 ± 1.41% *n* = 37 *vs* BaP + MV 28.34 ± 2.08% *n* = 41, *p* < 0.0001; Figures 3B,C), indicating that MV prevents the actin dynamics from damage induced by BaP exposure.

### MV Maintains Juno Levels in Oocytes of BaP-Exposed Mice

**FIGURE 4:**
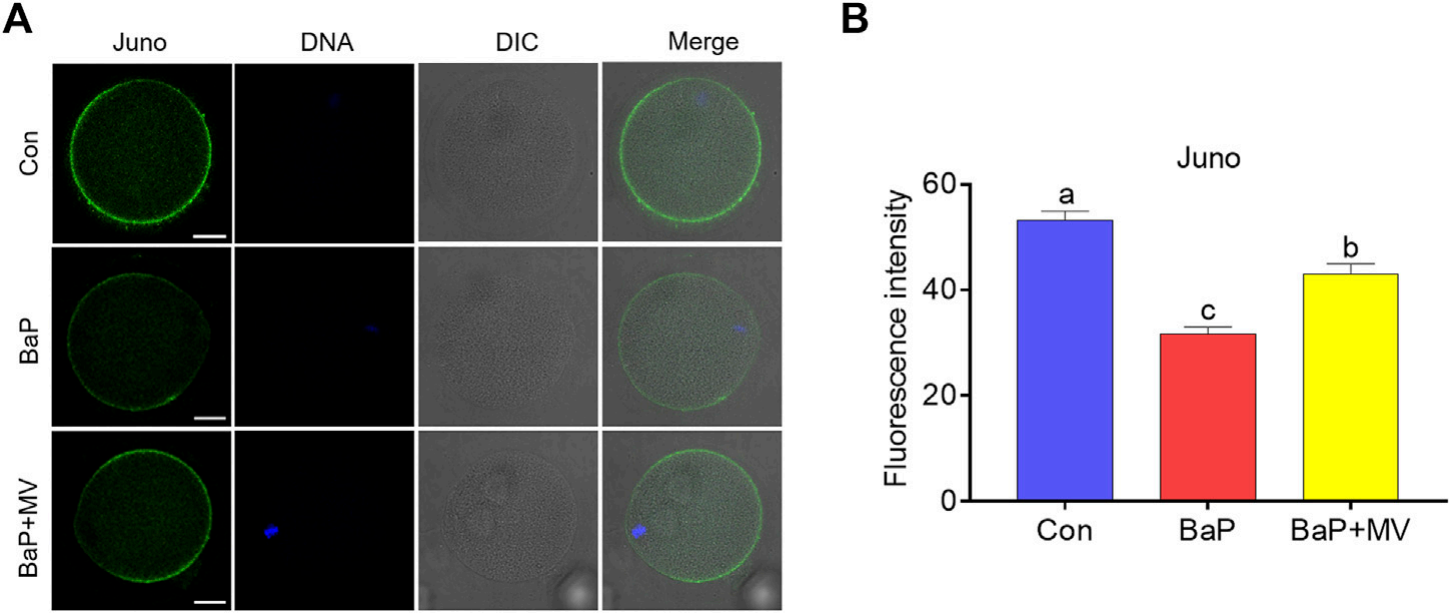
Effects of MV on Juno protein levels and distribution in the oocytes of BaP-exposed mice. **(A)** Representative images of Juno fluorescence signals and protein location in Con, BaP and BaP + MV oocytes. **(B)** The fluorescence intensity of Juno in the Con, BaP and BaP + MV groups. Scale bar = 25 μm. The data are presented as the mean ± SEM. ^a-c^ Means not sharing a common superscript are significantly different (*p* < 0.05).

Juno is a receptor on the membrane that binds the sperm head to mediate sperm-egg fusion. We next examined Juno levels on oocyte membranes by staining with FR4 antibody. As shown in Figures 4A,B, we found that the fluorescence intensity of Juno in BaP-exposed oocytes was lower than that in the Con group (Con 53.17 ± 1.75% *n* = 87 *vs* BaP 31.62 ± 1.35% *n* = 60, *p* < 0.0001), but it increased in the BaP + MV group compared to the BaP group (BaP 31.62 ± 1.35% *n* = 60 *vs* BaP + MV 42.99 ± 1.92% n = 67 *p* < 0.0001). Therefore, MV maintains Juno levels on the oocyte membrane of BaP-exposed mice.

### MV Suppresses Oxidative Stress in Oocytes of BaP-Exposed Mice

**FIGURE 5:**
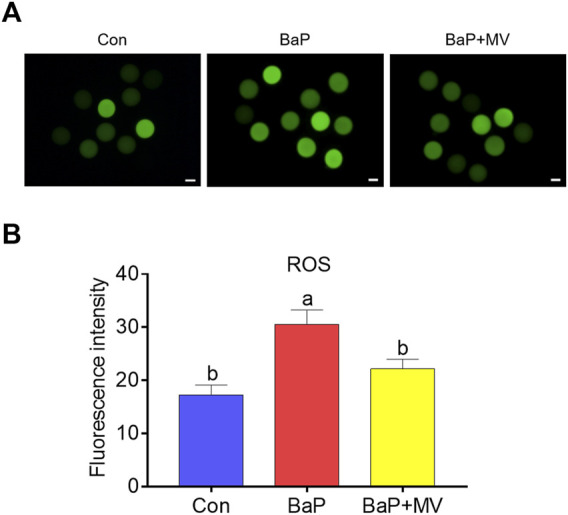
Effects of MV on ROS levels in the oocytes of BaP-exposed mice. Intracellular ROS content was measured using a dichlorodihydrofluorescein diacetate probe (DCFH-DA). **(A)** Representative images of ROS signals in Con, BaP and BaP + MV oocytes. **(B)** The fluorescence intensity of ROS in the Con, BaP and BaP + MV groups. Scale bar = 20 μm. The data are presented as the mean ± SEM. ^a-c^Means not sharing a common superscript are significantly different (*p* < 0.05).

To determine whether MV can alleviate oxidative stress induced by BaP exposure, we next assayed ROS levels with a DCFH-DA probe. As shown in Figures 5A,B, the fluorescence intensity of ROS was significantly increased in the BaP group compared to the Con group (Con 17.28 ± 1.84 *n* = 31 *vs* BaP 30.51 ± 2.74 *n* = 30, *p* < 0.001), and MV treatment effectively reduced the ROS levels in BaP-exposed oocytes (BaP 30.51 ± 2.74 *n* = 30 *vs* BaP + MV 22.15 ± 1.84 *n* = 31, *p* < 0.05), implying that MV alleviates oxidative stress in BaP-exposed oocytes.

### MV Inhibits DNA Damage and Apoptosis in Oocytes of BaP-Exposed Mice

**FIGURE 6:**
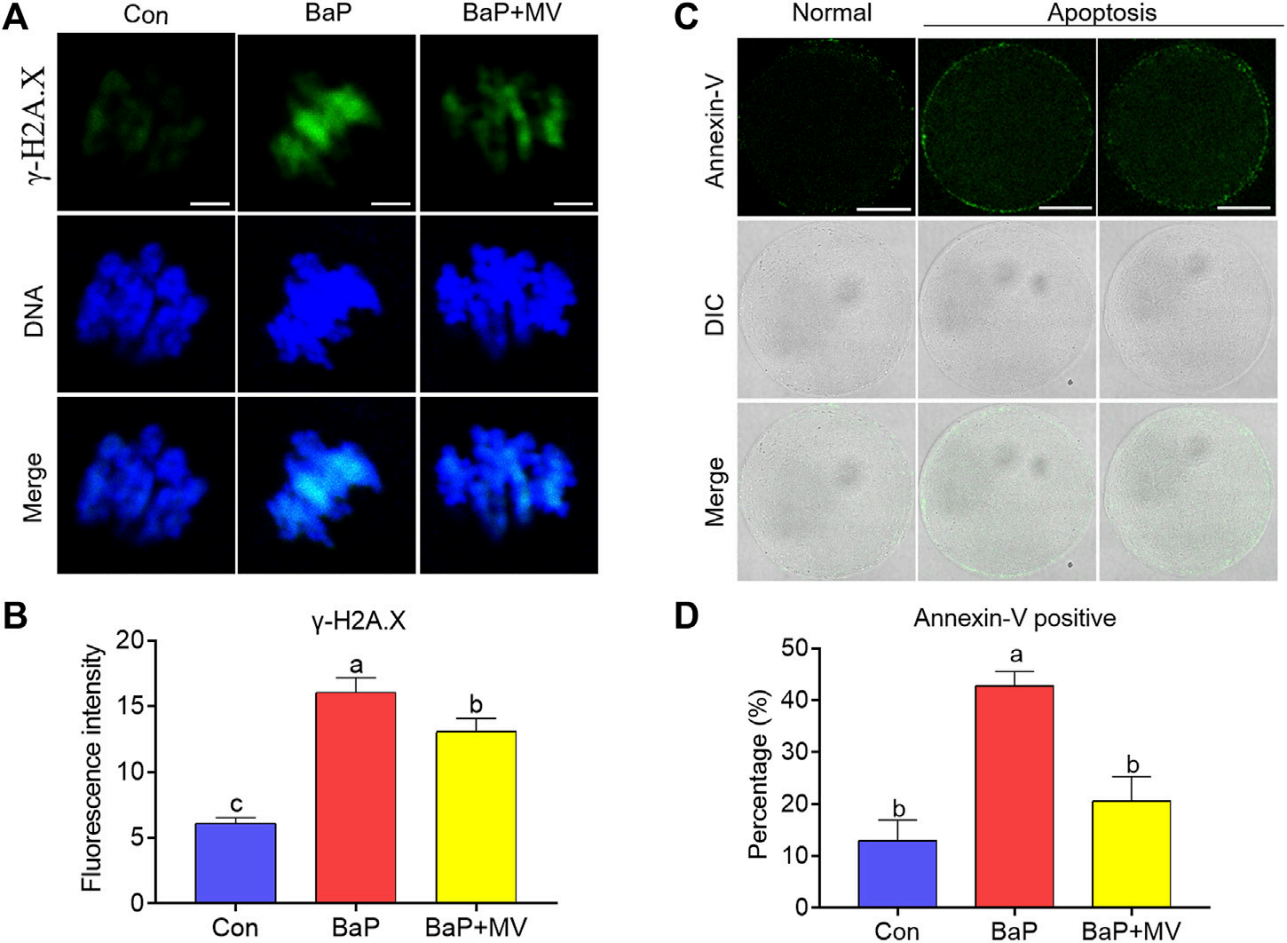
Effects of MV on DNA damage and apoptosis in the oocytes of BaP-exposed mice. DNA damage and early apoptosis in the oocytes were measured by staining with γ-H2A. X and Annexin-V probe, respectively. **(A)** Representative images of DNA damage in Con, BaP and BaP + MV oocytes. **(B)** The fluorescence intensity of DNA damage in the Con, BaP and BaP + MV groups. Scale bar = 5 μm. **(C)** Representative images of apoptosis in Con, BaP and BaP + MV oocytes. **(D)** The fluorescence intensity of apoptosis in the Con, BaP and BaP + MV groups. Scale bar = 50 μm. The data are presented as the mean ± SEM. ^a-c^Means not sharing a common superscript are significantly different (*p* < 0.05).

We stained oocytes with γ-H2A.X and Annexin-V probes to determine DNA damage and early apoptosis, respectively. As shown in Figures 6A,B, we observed that the fluorescence intensity was increased in the BaP group in comparison with the Con group (Con 6.12 ± 0.43 *n* = 99 *vs* BaP 16.08 ± 1.09 *n* = 89, *p* < 0.0001), while supplementation with MV markedly decreased the fluorescence intensity in the BaP + MV group (BaP 16.08 ± 1.09 n = 89 *vs* BaP + MV 13.08 ± 1.01 *n* = 74, *p* < 0.05), suggesting that MV can alleviate DNA damage in BaP-exposed oocytes. As shown in Figures 6C,D, the rate of apoptosis was higher in the BaP group than in the Con group (Con 12.92 ± 3.98% *n* = 47 *vs* BaP 42.77 ± 2.80% *n* = 49, *p* < 0.01), but lower in the BaP + MV group than in the BaP group (BaP 42.77 ± 2.80% *n* = 49 *vs* BaP + MV 20.59 ± 4.70% *n* = 53, *p* < 0.05), suggesting that MV can inhibit early apoptosis induced by BaP exposure.

## Discussion

In this study, we explored how MV protects oocytes from BaP exposure-mediated meiotic defects using a mouse model. Our findings showed that BaP exposure inhibits oocyte maturation and early embryonic development, associated with defective cytoskeletal dynamics and decreased Juno contents. Additionally, BaP induces excessive ROS accumulation, which consequently results in DNA damage and early apoptosis in oocytes. As expected, MV alleviated the abovementioned defective parameters induced by BaP exposure, thereby enhancing oocyte competence.

Our findings demonstrated that female mice exposed to BaP by gavage exhibited decreased oocyte maturation and subsequent embryo developmental capacity. Our studies further confirm previous observations that *in vivo* or *in vitro* BaP exposure remarkably inhibits oocyte maturation in mice and pigs, respectively (Zhang M. et al., 2018; Miao et al., 2018). Then, the BaP-exposed mice were treated with MV, and the decline in the oocyte maturation rate, as well as the 2-cell and blastocyst rates, were restored. As first polar body extrusion and embryonic development potential are indicators of oocyte nucleic and cytoplasmic maturation (Nie et al., 2020), respectively, MV can alleviate the decline in nucleic and cytoplasmic maturation in BaP-exposed oocytes.

Cytoskeletal structures such as microtubules and actin filaments play essential roles in oocyte meiotic progress (Duan and Sun, 2019). Normal spindle assembly is a premise for correct chromosome alignment and separation, whereas normal spindle formation depends on stable tubulin at an appropriate acetylation level (Xu et al., 2017). To further determine how MV enhances nucleic maturation in BaP-exposed oocytes, microtubule dynamics, α-tubulin acetylation levels and actin filaments were assayed. We found that spindle assembly and chromosome alignment were disrupted by BaP exposure and associated with decreased acetylation levels at α-tubulin. However, MV could notedly alleviate the defects of cytoskeletal structures in BaP-exposed oocytes, further explaining the ways MV ameliorates the decline of oocyte nucleic maturation (Yan et al., 2021).

We next observed that MV treatment could improve the proportion of 2-cell and blastocysts of BaP-exposed mice. Previous studies have reported that BaP exposure interferes with the gamete fusion process by perturbing the localization and protein level of Juno (Zhang M. et al., 2018). These defects in Juno protein might in part contribute to the reduction of consequent 2-cell and blastocyst development rates in BaP-exposed mice. Interestingly, we observed that MV could maintain the content and distribution of Juno protein on the oocyte membrane of BaP-exposed mice. Similarly, melatonin can stabilize Juno to promote fertilization ability in post-ovulary ageing mouse oocytes (Dai et al., 2017). As expected, we further found that MV treatment improved the proportion of 2-cell embryos and blastocysts in BaP-exposed mice. In addition, previous studies have demonstrated that MV can improve porcine oocyte *in vitro* maturation and protect oocytes from quality deterioration induced by postovulatory ageing or LPS exposure (Nie et al., 2019; Nie et al., 2020; Yan et al., 2021). As subsequent embryonic development potential is an indicator of oocyte cytoplasmic maturation, our findings show that MV might alleviate the deterioration of oocyte cytoplasmic maturation induced by BaP exposure.

We further found that BaP exposure caused excessive ROS accumulation, but MV treatment reduced ROS levels in the oocytes of BaP-exposed mice. MV is an excellent antioxidant with a very strong scavenging effect on ROS in cultured cells and tissues (Zhang X. et al., 2018; Liu et al., 2018). In addition, MV can also reduce excessive ROS accumulation in oocytes during *in vitro* maturation and the postovulatory ageing process or in LPS-exposed oocytes (Nie et al., 2019; Nie et al., 2020; Yan et al., 2021). Relatively low ROS levels prevent oocytes from impairments induced by oxidative stress. In this study, MV treatment reduced ROS levels in oocytes of BaP-exposed mice and thus avoided the induction of oxidative stress. A previous study revealed that melatonin and ginsenoside compound K can attenuate oxidative stress to protect porcine oocytes from meiotic defects caused by BaP exposure (Miao et al., 2018; Luo et al., 2020). In addition, we recently reported that MV inhibits the increase in ROS levels in porcine oocytes exposed to LPS (Yan et al., 2021). Oxidative stress results in diverse adverse changes, such as DNA damage and apoptosis in oocytes, which deteriorate oocyte quality and fertilization capacity (Tamura et al., 2008; Collins et al., 2015). Together with the findings of previous and current studies, MV is considered to protect oocytes from BaP exposure-mediated defects in BaP-exposed oocytes by attenuating oxidative stress, DNA damage and early apoptosis.

In summary, we found that MV might alleviate oocyte meiotic defects and quality deterioration in BaP-exposed mice. This at least in part contributes to the ROS scavenging property of MV. These findings provide new insight into reversing the adverse effects of BaP exposure on oocyte quality. However, the underlying mechanism still needs to be further explored.

## Data Availability

The original contributions presented in the study are included in the article/supplementary material, further inquiries can be directed to the corresponding authors.
